# Biomimetic Strategies for Bone Regeneration: Smart Scaffolds and Multiscale Cues

**DOI:** 10.3390/biomimetics11010012

**Published:** 2025-12-27

**Authors:** Sheikh Md Mosharof Hossen, Md Abdul Khaleque, Min-Su Lim, Jin-Kyu Kang, Do-Kyun Kim, Hwan-Hee Lee, Young-Yul Kim

**Affiliations:** Department of Orthopedic Surgery, Daejeon St. Mary’s Hospital, College of Medicine, The Catholic University of Korea, Daejeon 34943, Republic of Korea; mosharofkmu.dream@gmail.com (S.M.M.H.); abdulkhaleque.dream@gmail.com (M.A.K.);

**Keywords:** biomimetic scaffolds, vascularization, osteogenic activity, personalized bone repair

## Abstract

Bone regeneration remains difficult due to the complex bone microenvironment and the limited healing capacity of large defects. Biomimetic strategies offer promising solutions by using advanced 3D scaffolds guided by natural tissue cues. Recent advances in additive manufacturing, nanotechnology, and tissue engineering now allow the fabrication of hierarchical scaffolds that closely mimic native bone. Smart scaffold systems combine materials with biochemical and mechanical signals. These features improve vascularization, enhance tissue integration, and support better regenerative outcomes. Bio-inspired materials also help connect inert implants with living tissues by promoting vascular network formation and improving cell communication. Multiscale design approaches recreate bone nano- to macro-level structure and support both osteogenic activity and immune regulation. Intelligent and adaptive scaffolds are being developed to respond to physiological changes and enable personalized bone repair. This review discusses the current landscape of biomimetic scaffold design, fabrication techniques, material strategies, biological mechanisms, and translational considerations shaping next-generation bone regeneration technologies. Future directions focus on sustainable, clinically translatable biomimetic systems that can integrate with digital health tools for improved treatment planning.

## 1. Introduction

Critical-size bone defects represent injuries so extensive that they cannot heal on their own, creating a substantial clinical and socioeconomic burden [1]. These large defects disrupt normal regeneration, leading to prolonged disability, repeated surgical interventions, escalating healthcare costs, and marked declines in the patient’s quality of life. When the extent of bone loss exceeds the body’s intrinsic repair threshold, nonunion, instability, and long-term functional impairment frequently occur [2]. Common causes—including high-energy trauma, tumor excision, chronic infection, and congenital abnormalities—often produce segmental defects that surpass natural healing limits [3]. Patients with severe bone loss require complex reconstruction procedures and lengthened hospitalization, yet even with treatment, complications such as infection, graft failure, or amputation (reported in roughly 14.5% of cases) may still arise [4]. The economic burden is similarly substantial, with treatment expenses reaching nearly $300,000 per patient, while reimbursement rarely covers actual costs [4].

Traditional grafting methods provide important clinical options but have fundamental shortcomings. Autografts provide viable osteogenic cells and native biochemical cues, yet their use requires additional surgery and is limited by donor-site availability and morbidity [4]. Allografts are more readily available but may trigger immune responses, exhibit reduced biological activity, and pose a risk of disease transmission [5]. Synthetic materials, including bio-ceramics and degradable polymers, can be engineered for mechanical strength and shape but lack inherent bioactivity, often requiring exogenous signaling molecules to promote healing [6,7]. Despite decades of development, conventional materials still fall short of reproducing the dynamic, hierarchical, and mechanobiological nature of living bone tissue [5].

These limitations have propelled interest in biomimetics, an approach that derives design principles from the architecture, composition, and functional environment of native bone [8]. A biomimetic scaffold is conceived not as a passive filler but as an interactive microenvironment that integrates multiscale structure, biochemical patterning, mechanical responsiveness, and immune modulation [2]. Such engineered systems can coordinate osteogenesis and angiogenesis, regulate inflammatory transitions, and respond to mechanical loading in ways that influence cell behavior and molecular signaling [8]. Nonetheless, many existing models still do not capture the full three-dimensional complexity of the native bone niche, thereby reducing their predictive and translational relevance [9]. Vascular challenges are essential materials that degrade too rapidly, release bioactive components uncontrollably, or fail to engage immune–vascular interactions essential for healing [8]. Advances in bioinspired design, surface functionalization, and smart 3D fabrication now demonstrate that suitably engineered biomimetic scaffolds can promote osteogenesis, angiogenesis, and tissue integration—even without supplemental biological factors [10]. These developments highlight the potential of next-generation, multifunctional scaffolds that integrate hierarchical structural cues, adaptive mechanobiology, and immune-modulatory functions to overcome the persistent challenges of repairing critical-sized bone defects [9]. Consequently, a clear understanding of the biological complexity of bone healing, along with foundational knowledge of commercially available bone grafts, is essential for guiding the design of effective biomimetic strategies.

### 1.1. Purpose of This Review

This article provides a comprehensive overview of biomimetic approaches to the design of smart scaffolds inspired by native tissue architecture and (1) the multiscale biochemical, mechanical, and immunological cues that drive functional bone repair; (2) advanced fabrication technologies enabling multiscale biomimicry; (3) integration of cells, bioactive molecules, and ECM-based cues; and (4) translational pathways from laboratory systems to preclinical validation and early clinical readiness [11]. By evaluating current advancements and persistent limitations, we propose a rational framework for designing next-generation intelligent scaffolds capable of enabling personalized and clinically translatable bone repair [12].

### 1.2. Background

Bone is a dynamic, self-renewing tissue that serves a natural blueprint for biomimetic design. At the nanoscale, collagen fibrils interwoven with hydroxyapatite crystals create resilient composite that balances strength with flexibility. This hierarchical organization, spanning nano, micro, and macroscales, provides essential cues for cellular adhesion, differentiation, and matrix formation, making it a foundational model for the development of smart scaffolds in bone regeneration [13,14]. Mechanical and biochemical signaling in bone is tightly coupled, with cells sensing deformation, converting it into molecular cues, and adjusting matrix synthesis accordingly [15]. Regeneration also depends on coordinated immune and vascular activity: macrophage polarization shapes osteogenic signaling, while new vessels supply oxygen, nutrients, and progenitor cells. Biomimetic scaffolds aim to emulate this adaptive complexity by integrating structural, biochemical, mechanical, and immunological design cues [16]. Multiscale architectures support cell migration, nutrient exchange, and physiological load distribution, while advanced 3D/4D bioprinting enables spatial compartmentalization that mimics cortical–trabecular interfaces. Biochemical mimicry recreates ECM-like microenvironments through adhesive peptides, osteogenic proteins, angiogenic factors, and bioactive ions, establishing localized signaling niches that coordinate osteogenesis, angiogenesis, and mineralization in a time-dependent manner [17].

**Figure 1:**
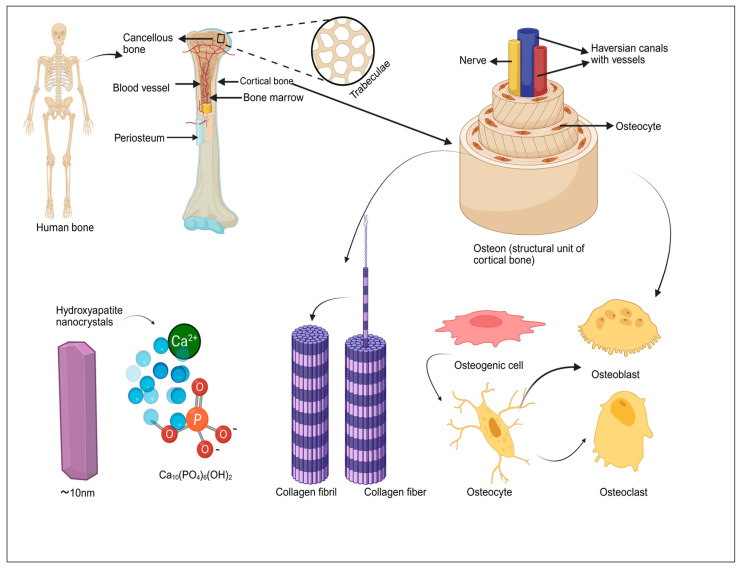
Hierarchical structure of human bone, from macroscopic features (cortical and cancellous bone, periosteum, and bone marrow) to microscopic components (trabeculae, osteons, HA nanocrystals, and collagen fibers) and the roles of key bone cells (osteogenic cells, osteoblasts, osteocytes, and osteoclasts).

Immune-focused design has become essential as osteo-immunomodulation is increasingly recognized as a key regulator of early inflammation, macrophage phenotype transitions, and downstream osteoblast–osteoclast activity [18]. This immune–mechanical synergy represents an emerging axis in scaffold innovation, linking inflammatory resolution with mechanical adaptation for improved integration [19]. Next, we outline the structural and functional principles that guide biomimetic scaffold design, followed by discussions of material platforms, fabrication technologies, and architectural strategies. Biomimetic systems can enhance bone formation, vascularization, and integration even without added biological factors [17]. Three-dimensionally printed calcium phosphate and hydroxyapatite scaffolds offer promising alternatives to grafts, though their intrinsic osteoinductivity remains limited [14,20]. This limitation underscores the need for cell-free, biologic-free constructs capable of modulating the local immune and biochemical milieu [7]. Although many degradable polymers and calcium phosphate ceramics exist, the incorporation of osteogenic cells or growth factors remains constrained by regulatory and safety concerns [14]. Consequently, osteo-immunomodulation has emerged as a practical means to guide early inflammation, promote osteogenesis, and synchronize bone remodeling. Macrophage transition from a pro-inflammatory to a pro-regenerative phenotype is now recognized as a critical determinant of successful healing [21]. Smart 3D scaffolds integrate multilevel cues with responsive materials that adapt to local biochemical and mechanical conditions [6]. Additive manufacturing, electrospinning, and bioprinting now enable precise control over architecture, porosity, and bioactivity, transforming biomimetic design into clinically relevant solutions [22]. Architectural strategies recreate bone’s hierarchical organization, from nanoscale mineral features to macroscale pore networks, enabling cell infiltration, nutrient transport, and vascular ingrowth while preserving mechanical stability [23]. Translational progress will rely on improving scalability, immunocompatibility, and regulatory compliance [24]. Immune modulation supports constructive remodeling by preventing chronic inflammation, while vascular cues promote early perfusion and long-term graft stability [25]. Integrating architecture, signaling, mechanics, and immunoregulation into a unified scaffold platform is driving the next generation of multifunctional implants [20]. Emerging evidence shows that these integrated cues can direct stem cell fate, accelerate angiogenesis, and align bone regeneration with immune resolution [26]. The hierarchical structure of bone, comprising cortical and cancellous bone, is essential for its mechanical strength and biological functionality. Cortical bone, with its densely packed lamellae and trabeculae aligned along stress directions, provides rigidity and toughness, while cancellous bone offers elasticity and adaptability, facilitating growth and repair. Dan Huang et al. elaborated that the hollow HA microsphere offers benefits in attracting calcium ions and modulating inflammatory responses to support bone regeneration and remodeling. Despite the significant advancements in biomimetic bone structure, design challenges persist. Additionally, the utilization of biomimetic strategies in scaffold design has introduced an innovative approach to bone tissue regeneration. Future efforts must enhance scaffold adaptability, personalize structural design, and harmonize fabrication workflows with regulatory standards [27]. Smart scaffolds that respond dynamically to local signals are expected to improve stability, remodeling, and clinical readiness [14] (Figure 1).

### 1.3. Biomimetic Scaffold Design Principles

The immune–vascular interplay defines the pace and quality of new bone formation, positioning native bone as a dynamic template for biomimetic scaffold design [28]. (i) Scaffolds must avoid cytotoxicity and minimize immunogenic reactions, with surface cues that promote osteoblast and mesenchymal stem cell (MSC) attachment for effective regeneration [29]. (ii) Controlled biodegradation is essential, ensuring that scaffold resorption matches the rate of new bone formation while generating only nontoxic by-products [30]. (iii) Mechanical requirements are met through engineered composite architectures optimized for load bearing and regeneration. (iv) A highly porous, fully interconnected architecture (≈60–90%) is crucial for nutrient transport, osteoblast migration, and vascular ingrowth [17]. Pore sizes of 100–300 µm support cellular infiltration, while ~300 µm pores strongly promote angiogenesis [14]. Modern 3D bioprinting platforms allow precise control over pore geometry and spatial organization, enabling architectural optimization for efficient remodeling [29]. (v) Bioactive scaffolds actively stimulate osteogenesis, integrating molecular cues rather than serving as passive matrices [31]. Strategies include incorporating growth factors, adhesive peptides, and mineralized coatings to enhance osteoinductivity and strengthen osteoconductive signaling [29]. (vi) Early and sustained vascularization is vital for graft survival and can be significantly improved through angiogenic factor delivery and hierarchical pore networks designed to facilitate rapid vessel ingrowth [28]. (vii) Material options range from natural and synthetic polymers to bioactive ceramics and composites, each offering specific advantages in biocompatibility, degradation control, and osteoconductive performance [28]. Effective biomimetic scaffolds replicate the hierarchical architecture of native bones by integrating macro-porosity, microchannels, and nanoscale topography to guide tissue ingrowth [32]. Their mechanical properties and degradation profiles must be tuned to match physiological loading and natural bone turnover [22]. Bone heals through a coordinated cascade of inflammation, vascularization, matrix deposition, and remodeling; therefore, mimicking this dynamic physiology requires scaffolds that deliver biochemical and mechanical cues in a temporally controlled manner [33]. Multiscale biomimicry is achieved by combining material chemistry, topographical guidance, and the controlled release of osteogenic signals to recreate the bone microenvironment [34]. Smart fabrication technologies now enable spatially patterned constructs that respond adaptively to cellular activities, allowing the precise placement of structural features, signaling molecules, and, when needed, living cells [35]. Biomimetic scaffolds represent a shift from passive structural supports to active, instructive systems capable of guiding tissue repair [36]. Recent studies show that designs integrating hierarchical architecture with timed biochemical cues enhance osteogenesis, promote angiogenesis, and improve host integration compared with conventional scaffolds [37]. By replicating bone’s structural, biochemical, and mechanical complexity, biomimetic strategies direct cell behavior more effectively [38]. This schematic highlights how biomimetic scaffold design draws inspiration from the hierarchical organization of native bone. At the macroscopic level, cortical bone provides structural rigidity, while the microscopic osteon system guides nutrient exchange and cellular communication. At the nanoscale, hydroxyapatite crystals supply the mineral template essential for osteogenic signaling. Smart scaffolds emulate these multi-level features through controlled porosity, optimized pore architecture, and nanoscale bioactive cues, collectively enhancing osteoblast adhesion, differentiation, and matrix formation. By integrating structural, compositional, and cellular-scale elements, biomimetic scaffolds more effectively recapitulate the regenerative microenvironment required for functional bone repair.

**Figure 2:**
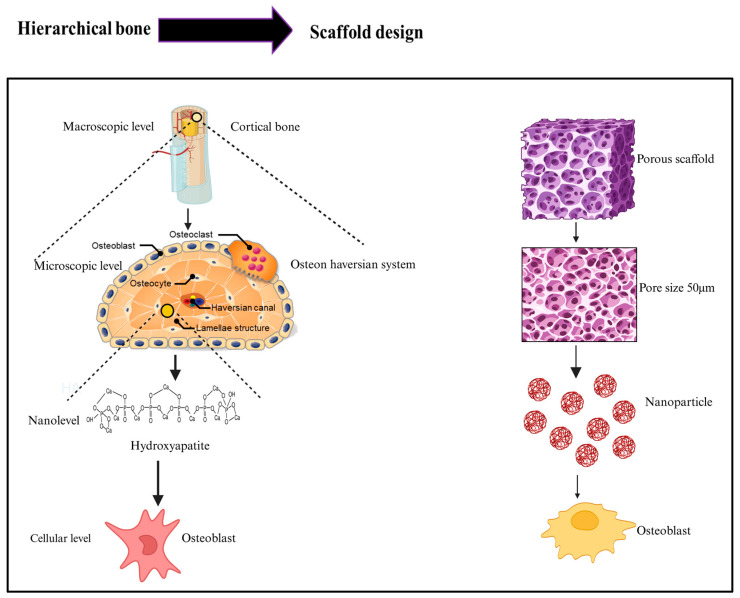
Left panel illustrates the natural bone hierarchy as follows: at the macroscopic level, cortical bone contains blood vessels; at the microscopic level, the osteon (Haversian system) consists of osteoblasts, osteocytes, osteoclasts, Haversian canals, and lamellar structures; at the nanolevel, hydroxyapatite (HA) provides mineral support; and at the cellular level, osteoblasts maintain bone formation. The right panel demonstrates the biomimetic scaffold design strategy as follows: a porous scaffold mimics the macrostructure; pore size tuning recreates microscopic architecture; and the incorporation of nanoparticles at the nanoscale promotes osteoblast activity, collectively aiming to replicate the hierarchical features and functional behavior of native bone tissue.

Emerging directions highlight the development of intelligent scaffolds that synergistically integrate mechanobiological cues, immune modulation, and vascular patterning for sustained and predictable bone regeneration [31]. Smart materials will enable real-time responsiveness to local biochemical and mechanical stimuli [39]. Ultimately, the convergence of biomimicry with advanced 3D fabrication is expected to drive a new generation of intelligent scaffolds capable of supporting precise and durable tissue regeneration [11]. These innovations position biomimetic systems as key components in the future translation of personalized bone repair [25] (Figure 2).

## 2. Integrated Biomimicry Framework in Bone Tissue Engineering

A biomimicry framework in bone tissue engineering focuses on recreating the multiscale architecture and functional behavior of native bone by integrating structural, biochemical, mechanical, immune, and vascular cues into scaffold design [22]. These goals align with core principles required for clinically successful regeneration, including biocompatibility, controlled degradation, mechanical competence, spatial organization, osteoinductivity, vascularization, and appropriate material selection [28].

### 2.1. Structural Biomimicry

Structural biomimicry is central to effective bone regeneration because native bone exhibits a hierarchical organization spanning mineralized collagen fibrils at the nanoscale, lamellar osteons, and trabecular lattices, and dense cortical shells at the macroscale [40]. Replicating this multilevel architecture guides cell migration, facilitates oxygen and nutrient transport, and distributes mechanical loads efficiently. Biomimetic smart scaffolds therefore integrate nanoscale features such as hydroxyapatite nanocrystals or collagen-mimetic motifs to enhance osteoblast adhesion and stimulate mineralized matrix formation [41]. At larger scales, gradient porosity that transitions from compact cortical-like regions to open trabecular-like domains provides a balance of mechanical strength with cellular infiltration and vascular ingrowth [42]. Aligned microchannels further direct the movement of osteoblasts and endothelial cells migration, while hierarchical surface roughness mimicking the ECM activates integrin-mediated signaling pathways [30]. Advances in high-resolution 3D printing, electrospinning, and hybrid fabrication now enable precise control over these multiscale structural cues, leading to significantly improved osteo-conduction. Furthermore, they are effective on these structural gradients, resulting in markedly improved osteo-conduction and effective bridging of critical-size bone defects in preclinical studies [40]. Importantly, such scaffolds must remain non-cytotoxic, minimize inflammatory responses, and provide a stable interface for mesenchymal (MSC) and osteoblast adhesion [1]. This architectural foundation underpins the coordinated integration of biochemical, mechanical, and vascular cues, highlighting the significance of structural biomimicry within smart, multiscale scaffold strategies for robust bone regeneration [30].

### 2.2. Biochemical Biomimicry

The biochemical microenvironment of bone consists of ECM proteins, adhesive peptides, bioactive ions, and spatiotemporally controlled growth factor gradients [32]. Biomimetic scaffolds replicate these molecular signals by incorporating ECM-derived ligands such as RGD, GFOGER, or osteopontin mimetic sequences that enhance cell adhesion spreading and osteogenic differentiation [43]. Substitution of osteogenic ions Mg^2+^, Sr^2+^, and Si^4+^ accelerates bone formation, modulates macrophage activity, and contributes to immune homeostasis [44]. Controlled and sequential release of BMP-2, VEGF, or SDF-1 mimics the natural timing of osteoinduction angiogenesis and progenitor recruitment observed during bone healing. Mineralization-inspired surface coatings further promote hydroxyapatite nucleation and strengthen interfacial bonding with host bone [41]. Biochemical mimicry is closely coupled with controlled biodegradation as follows: scaffold resorption must be synchronized with new bone formation, producing only nontoxic by-products while sustaining the presentation of molecular cues throughout regeneration [41,45].

### 2.3. Mechanical Biomimicry

Bone is a mechanosensitive tissue regulated by dynamic loading. Biomimetic scaffolds therefore reproduce the mechanical environment essential for osteogenic differentiation and matrix maturation. This includes stiffness gradients representative of cortical and cancellous regions, viscoelastic and fatigue behaviors matching physiologic bone dynamics, and architectures designed to distribute load naturally and prevent stress shielding [46]. Emerging piezoelectric and mechanoresponsive materials convert mechanical deformation into localized electrical or biochemical signals, directly stimulating osteoblast activity through mechano-transduction pathways [45]. These mechanically tuned constructions have demonstrated superior performance in both dynamic in vitro systems and in vivo load-bearing models [47].

### 2.4. Vascular Biomimicry

Effective bone repair is dependent on rapid and sustained vascularization, which ensures nutrient delivery, waste removal, and recruitment of osteoprogenitor and immune cells [48]. Biomimetic strategies replicate natural vascular architecture through prefabricated microchannels, vascular conduits produced via sacrificial templating approaches, and hierarchical pore networks that guide vessel ingrowth [49]. Angiogenic factor gradients emulate early sprouting and maturation phases of neovascularization, while endothelial-MSC co-culture systems generate pre-vascularized networks capable of fusing with host vasculature upon implantation. Oxygen-releasing materials further maintain early graft viability during periods of limited perfusion [50]. These vascular features align with the broader principle that a scaffold must integrate angiogenic cues and structural porosity, especially pores of ~300 µm, to support robust vessel ingrowth and accelerate defect repair [51].

### 2.5. Unified Biomimetic Design and Material Selection

Material selection acts as a unifying element of the biomimicry framework. Natural polymers, synthetic polymers, bioactive ceramics, and composite systems each provide distinct advantages in biocompatibility, biodegradation control, mechanical behavior, and osteoconductivity [52]. Tailoring these materials enables scaffolds to harmonize structural, biochemical, mechanical, immune, and vascular functions within a single platform [53].

Together, these interconnected principles produce a biomimetic strategy that advances bone tissue engineering beyond traditional scaffold designs. By integrating hierarchical architecture, physiologically relevant biochemical signaling, mechanically adaptive behavior, immunomodulation, and vascular guidance, modern biomimetic scaffolds more closely emulate native bone’s complexity and provide a robust foundation for regenerating critical-size defects [54].

### 2.6. Osteo-Immunomodulatory Mechanisms in Biomimetic Bone Regeneration

**Figure 3:**
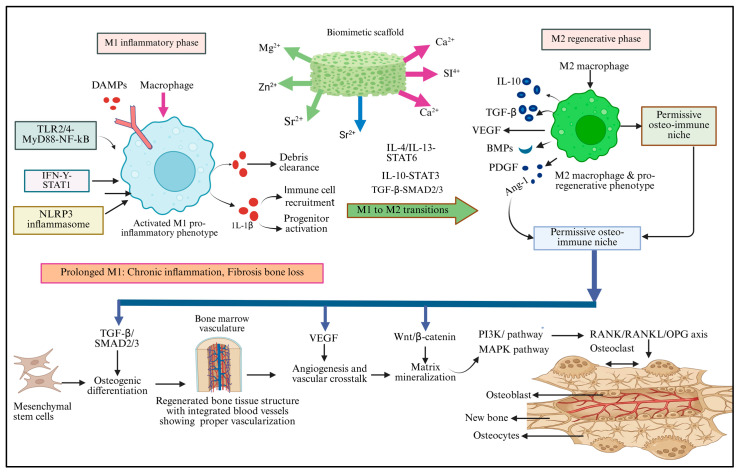
Schematic representation of osteo-immunomodulatory pathways in biomimetic scaffold-mediated bone regeneration-bioactive ion release (Mg^2+^, Zn^2+^, Sr^2+^, Si^4+^, and Ca^2+^) from scaffolds orchestrates macrophage polarization dynamics. M1 macrophages (left, red) respond to damage signals through TLR2/4–NF-κB, IFN-γ–STAT1, and NLRP3 pathways, secreting TNF-α and IL-1β for debris clearance and immune recruitment. Prolonged M1 activation causes pathological inflammation (warning box). Osteo-immunomodulatory scaffolds drive M1-to-M2 transition (center) via IL-4/IL-13–STAT6, IL-10–STAT3, and TGF-β–SMAD2/3 signaling. M2 macrophages (right, green) establish a permissive osteo-immune niche, secreting IL-10, TGF-β, VEGF, BMPs, PDGF, and Ang-1. These factors activate downstream pathways (bottom, purple) as follows: TGF-β/SMAD2/3 drives MSC osteogenic differentiation, VEGF enhances angiogenesis, Wnt/β-catenin promotes mineralization, PI3K/Akt and MAPK integrate mechanical-biochemical signals, and RANK/RANKL/OPG regulation ensures balanced bone remodeling. The outcome is regenerated, vascularized bone tissue with coordinated osteoblast-osteoclast coupling.

Bone regeneration represents a tightly orchestrated, immune-guided process wherein biomimetic scaffolds function as active regulators of macrophage behavior rather than passive structural supports, with controlled release of bioactive ions (Mg^2+^, Zn^2+^, Sr^2+^, Si^4+^, and Ca^2+^) serving as a unifying mechanism that integrates immune regulation, vascularization, and osteogenesis into a synchronized regenerative cascade [55]. Upon scaffold implantation or bone injury, macrophages detect damage-associated molecular patterns, extracellular matrix cues, and scaffold-derived ions, triggering a transient M1 pro-inflammatory phenotype through TLR2/4–MyD88–NF-κB, IFN-γ–STAT1, and NLRP3 inflammasome signaling pathways, with resulting TNF-α and IL-1β secretion facilitating essential early-stage functions including debris clearance, immune cell recruitment, progenitor activation, and initial osteoclast genesis [4]. However, prolonged M1 activation sustains NF-κB–TNF-α–IL-1β signaling that inhibits osteoblast differentiation and promotes pathological outcomes including chronic inflammation, fibrotic encapsulation, and bone loss, a challenge addressed by osteo-immunomodulatory scaffolds that selectively attenuate excessive inflammatory signaling while preserving beneficial early immune responses and driving a timely M1-to-M2 transition through the activation of IL-4/IL-13–STAT6, IL-10–STAT3, and TGF-β–SMAD2/3 signaling axes [56]. STAT6-polarized M2 macrophages establish a permissive osteo-immune niche by secreting IL-10, TGF-β, VEGF, BMPs, PDGF, and Ang-1, which simultaneously suppress inflammation, enhance angiogenesis, and activate multiple downstream pathways as follows: TGF-β/SMAD2/3 signaling drives osteogenic commitment and extracellular matrix synthesis in mesenchymal stem cells, VEGF establishes immune–vascular crosstalk enhancing neovascularization and osteoprogenitor recruitment, Wnt/β-catenin activation promotes matrix mineralization while suppressing excessive osteoclast activity, and PI3K/Akt and MAPK pathways integrate mechanical cues from scaffold architecture with biochemical signals from bioactive ions [57]. Macrophage-mediated regulation of the RANK/RANKL/OPG axis ensures balanced osteoblast coupling and coordinated bone remodeling, preventing both insufficient resorption and excessive bone loss [58]. Through these interconnected osteo-immunomodulatory pathways, bioactive ion-functionalized scaffolds transform the host inflammatory response from a potential barrier into a regenerative driver, synchronizing inflammation resolution, vascularization, redox balance, and osteogenesis to enable stable, functional, and long-term bone regeneration-a paradigm shift from passive structural support to active immune regulation that represents a fundamental advance in regenerative medicine strategies for bone repair [59] (Figure 3).

## 3. Fabrication Technologies

Biomimetic bone regeneration strategies employ smart scaffolds that integrate multiscale structural, mechanical, and biological cues to recapitulate native bone architecture and function. Advanced fabrication techniques provide precise spatial control, enabling the translation of nanoscale extracellular matrix mimetic features into macroscale load-bearing geometries. In this context, emerging additive manufacturing technologies have facilitated the development of biodegradable polymer scaffolds reinforced with carbon-based materials, allowing tailored architectural design, enhanced mechanical performance, and improved biological functionality [60]. Three-dimensional printing methods including material extrusion techniques such as fused deposition modeling (FDM) and direct ink writing (DIW), powder bed fusion processes such as selective laser sintering (SLS) and selective laser melting (SLM), and vat photopolymerization approaches such as stereolithography (SLA) and digital light processing (DLP) enable the fabrication of patient-specific scaffolds with precisely tunable porosity and mechanical properties. The inclusion of digital light processing (DLP)-based vat photopolymerization, along with permeability assessment and foam or triply periodic minimal surface (TPMS) scaffold architectures, is important because these approaches directly address key structure function relationships in biomimetic bone regeneration, while permeability analysis provides quantitative insight into mass transport and vascularization potential [61]. In parallel, electrospinning produces nanoscale fibrous matrices that mimic extracellular matrix architecture and enhance cell adhesion and signaling. For biodegradable ceramic scaffolds, which do not readily melt, additive manufacturing is predominantly achieved using powder bed fusion techniques, particularly SLS. To overcome the intrinsic brittleness of ceramics, these materials are commonly combined with biodegradable polymers, thereby improving toughness and printability. Consequently, hybrid material systems are often processed using alternative techniques such as DIW, enabling better structural integrity and biological performance [62]. FDM has shown rapid development in recent years due to its simplicity, speediness, and large-scale rate of production. Raw materials in FDM are filaments that are partially melted by a heater and extruded from a nozzle. In the case of DIW, the material used is colloidal ink, which is directly extruded through an orifice or nozzle without heating. These inks can maintain their shape during solidification. (ii) Powder bed fusion: SLS and SLM are categorized as powder bed fusion technologies since they utilize thermal energy to selectively melt powder materials of a powder bed. The raw material is typically in the form of powder-based particles for these AM-based technologies. Complete melting is achieved in SLM, while in SLS heat provokes material fusion at the molecular level instead of complete melting stereolithography (SLA), and selective laser sintering (SLS) enable customized geometries, pore gradients, and mechanical tuning [17,63]. These approaches translate digital designs into patient-specific scaffolds with reproducible fidelity [34]. Electrospinning generates nanoscale fibrous networks that replicate extracellular matrix topography and enhance cell attachment and signaling [64]. These strategies are evaluated using in vitro cell culture systems, dynamic mechanobiological platforms, and in vivo critical-size bone defect models to assess osteogenesis, vascularization, and functional integration. Composite systems combining biodegradable polymers, ceramics, hydrogels, and bioactive additives are used to achieve mechanical competence, osteoinductivity, and controlled degradation. Shape-memory and responsive materials enable adaptive behavior under physiological stimuli. Hybrid systems combining electrospinning with 3D printing improve hierarchical integration across scales [65]. Bioprinting introduces living cells, bioinks, and growth factors directly into the scaffold, creating biologically active constructs capable of self-organization and vascular invasion [66]. Smart, multiscale scaffolds significantly enhance cell–material interactions, promote vascularized bone formation, and improve mechanical stability. The integration of AI-guided design further accelerates optimization and personalization, highlighting strong translational potential for predictable and long-lasting bone regeneration. Four-dimensional printing advances this further by embedding shape-memory or stimuli-responsive materials, enabling dynamic adaptation to physiological cues such as stress, temperature, or pH. AI-guided design now supports data-driven optimization of scaffold parameters, predicting cell-material interactions and mechanical behavior to accelerate personalized regenerative solutions [67].

### 3.1. Contribution of Fabrication Technologies to Biomimicry

The advancement of biomimetic scaffold design is closely linked to progress in fabrication technologies that allow precise manipulation of structure, mechanics, and biological functionality. Modern platforms have evolved from simple porous scaffolds to digitally engineered, multi-material constructs capable of reproducing bone’s hierarchical architecture and functional complexity [68]. This section outlines key fabrication modalities and their contributions to biomimetic bone regeneration [69] (Table 1).

### 3.2. Three-Dimensional Printing Technologies

**Figure 4:**
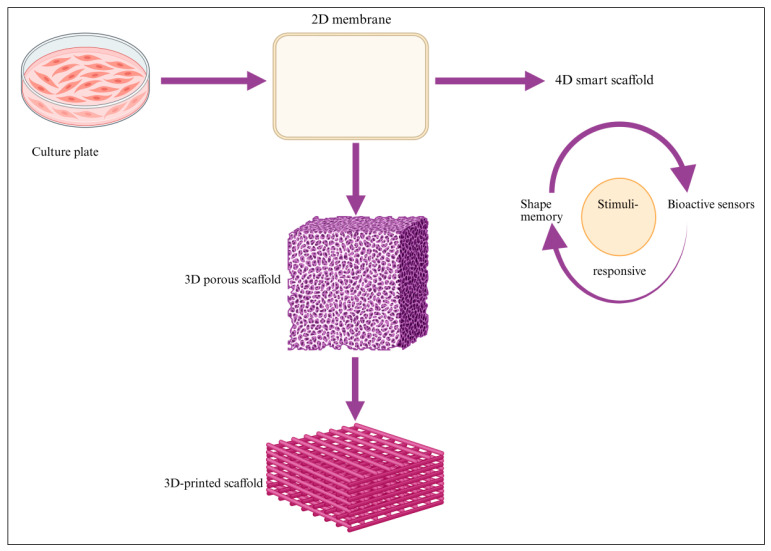
This figure illustrates the progressive transition from conventional cell culture systems to engineered 2D membranes and structured 3D scaffolds. These platforms enable improved spatial organization, mechanical support, and biomimetic microenvironments. Integration of stimuli-responsive and shape-memory features marks the advancement toward 4D smart scaffolds capable of dynamic, adaptive functionality.

Biomimetic bone regeneration relies on smart scaffolds that integrate structural, mechanical, and biological cues across multiple length scales to emulate native bone organization. Additive manufacturing enables precise spatial control over scaffold geometry, pore architecture, mechanical behavior, and patient-specific shape, supporting the fabrication of functionally graded and customized constructs for regenerative applications [10]. There are three classified 3D printings, such as inkjet bioprinting, extrusion-based bioprinting, and laser-assisted bioprinting. Inkjet bioprinting operates by depositing droplets of bioink in a non-contact, layer-by-layer manner [70]. It provides medium printing resolution and is compatible with natural polymers, synthetic polymers, and cell suspensions. Cell viability is generally moderate due to thermal or piezoelectric stresses during droplet ejection. Inkjet bioprinters are low to medium in cost and offer advantages such as versatility, simplicity, and low operational cost [71]. However, this technique is limited by low bioink viscosity requirements, which restricts structural stability and makes it difficult to build mechanically robust three-dimensional constructs [17]. Extrusion bioprinting is the most widely used technique and relies on continuous deposition of bioinks through pneumatic, piston-driven, or screw-driven systems. It offers medium resolution and supports a wide range of natural and synthetic polymers, including highly viscous and cell-laden bioinks [24]. The printing speed is relatively slow, but cell viability ranges from medium to high, depending on extrusion pressure and nozzle diameter. Printer costs vary from low to high, depending on system complexity. Key advantages include the ability to print multiple materials and complex compositions. Major limitations are lower printing accuracy compared to other methods and restricted use of delicate or low-viscosity biomaterials. Laser-assisted bioprinting is a nozzle-free technique that uses focused laser pulses to precisely transfer bioink droplets onto a substrate. It provides high spatial accuracy, although effective resolution is typically described as low to medium at the construct scale [72]. This method primarily prints cells suspended in liquid media and achieves high cell viability due to the absence of mechanical stress. Printing speed is moderate, while equipment cost is high. The main advantages include high precision and single-cell-level manipulation. For large animals, a 3D skin printing system equipped with a three-head dispenser and an integrated 3D scanner was used. Wound sizes of 40 × 40 mm (up to 100 × 100 mm) were automatically scanned, and printing was performed immediately after scanning. For small-animal experiments, a dedicated 3D skin printing system was employed using four types of composite hydrogel as bioinks. Experiments were conducted on rats with round skin defects measuring 3 cm in diameter. The resulting 3D-printed skin constructs demonstrated complete epidermal coverage (100%) at a scale of 1 mm. The bioink, composed of collagen, hyaluronic acid, and fibrin, supported fibroblasts and keratinocytes, and tissue organization was confirmed by Masson’s trichrome staining. However, the need for low viscosity bioinks limits vertical build-up, making the fabrication of large 3D structures challenging. To overcome such limitations, electrospinning is often employed to fabricate fibrous scaffolds by ejecting polymer solutions under high electric fields [73]. This technique achieves high resolution at the nanoscale, producing fibers with diameters comparable to the native extracellular matrix. Both natural and synthetic polymers can be processed, and the method allows relatively fast fabrication. However, cell viability cannot be maintained during electrospinning, as cells are typically not incorporated directly, and printer costs can be moderately high. The main advantages include nanoscale pore formation and a high surface area, which promotes cell adhesion and scaffold integration [74]. The primary limitation is poor control over macroscopic shape and complex 3D geometry. Three principals of 3D printing modalities are widely applied in bone tissue engineering and those are extrusion-based printing (e.g., fused deposition modeling, FDM), inkjet printing, and stereolithography (SLA) [34]. Among these, FDM is extensively used due to its ability to process FDA-approved thermoplastic polymers such as polycaprolactone (PCL) and poly (lactic-co-glycolic acid) (PLGA). FDM produces interconnected macroporous scaffolds with programmable stiffness and controlled biodegradation, making it suitable for load-bearing and large bone defects [75]. Studies demonstrate that FDM-fabricated scaffolds provide adequate mechanical strength, promote cell infiltration, and support osteogenic differentiation when combined with appropriate surface modifications or bioactive additives [76]. The reproducibility and scalability of this technique further enhance its translational potential. FDM extrudes thermoplastic polymers (e.g., PCL, PLGA) layer by layer, generating interconnected macroporous networks with programmable stiffness and biodegradation [9]. Advantages include cost-effectiveness, good mechanical strength, scalability for large defects, and available FDA-approved polymers. Despite these advantages, FDM offers lower spatial resolution compared with photopolymer-based techniques and has limited compatibility with cell-laden bioinks, restricting its ability to directly incorporate living cells during fabrication. Overall, extrusion-based additive manufacturing represents a robust and clinically relevant platform for developing biomimetic bone scaffolds. When integrated with multiscale design principles and complementary fabrication strategies, these systems contribute significantly to the advancement of predictable and patient-specific bone regeneration [77] (Figure 4).

Stereolithography (SLA)

This strategy employs high-resolution fabrication to reproduce bone-like architecture across length scales, integrating structural precision with tunable mechanical and biological properties. Light-based printing enables accurate control over pore size, microchannels, and stiffness, supporting biomimetic designs that guide cell organization and tissue maturation [17,78]. Stereolithography (SLA) is a vat-photopolymerization method that uses focused UV or visible light to selectively cross-link photosensitive resins in a layer-by-layer manner. SLA achieves sub-50 µm resolution and enables the fabrication of finely detailed geometries suitable for trabecular- and cortical-bone analogs. The technique is particularly effective for producing complex microchannel networks and hydrogel-based constructs, making it highly attractive for soft tissue and vascularized interfaces with high spatial accuracy [79]. SLA-fabricated scaffolds exhibit excellent geometric fidelity, uniform pore architecture, and tunable stiffness through resin chemistry. These features support precise cell patterning, enhanced nutrient transport, and controlled mechanobiological signaling, which are critical for guiding osteogenesis and vascular ingrowth. The advantages of these techniques include high precision, tunable stiffness through resin chemistry, and suitability for fabricating complex microchannel networks [80]. Photopolymer resins require careful biocompatibility assessment because residual photo initiators or unreacted monomers may compromise cell viability. Post-processing steps such as washing and secondary curing are often necessary, increasing fabrication time and effort. Nonetheless, SLA offers a powerful platform for designing biomimetic bone scaffolds with unmatched precision and architectural control. Future research directions focus on developing bioresorbable and cell-friendly photopolymers, integrating SLA with bioprinting or hybrid fabrication approaches, and expanding its application from soft tissues toward mechanically competent, vascularized bone regeneration systems. Photopolymers often require thorough biocompatibility validation and additional post-processing steps [81]. This approach enables layer-by-layer UV/light exposure to induce hydrogel cross-linking, making it highly suitable for fabricating soft tissue constructs with high accuracy [82].

Selective Laser Sintering (SLS)

This biomimetic strategy focuses on reproducing the structural and mechanical functions of native bone by combining precise architectural control with mechanically competent materials. Additive manufacturing enables the fabrication of scaffolds that replicate bone-like load distribution, porosity, and spatial organization across multiple length scales [34]. SLS is a powder bed fusion technique that uses a high-energy laser to sinter ceramic, metallic, or composite powders without the need for binders. SLS is particularly suitable for producing strong, load-bearing scaffolds using materials such as titanium and hydroxyapatite/tricalcium phosphate (HA/TCP) composites. The technique offers high geometric fidelity and is well-suited for large or mechanically demanding bone defects. SLS-fabricated scaffolds exhibit excellent mechanical strength, high-dimensional accuracy, and well-interconnected porosity, ensuring structural stability and efficient load transfer. These properties make SLS constructs especially valuable for applications requiring immediate mechanical support [83]. Despite its advantages, SLS is associated with high equipment- and processing costs and is limited to materials that can withstand laser sintering conditions, restricting material selection and biofunctionalization options. Collectively, SLS and complementary 3D printing approaches enable structural, mechanical, and architectural biomimicry, forming the foundation of modern scaffold engineering. Future directions include integrating bioactive phases, improving material versatility, and combining SLS with surface modification or hybrid fabrication strategies to enhance biological performance while maintaining mechanical robustness. High strength for large or load-bearing defects are methods suitable for titanium or HA/TCP composites but are limited by high cost, material compatibility with laser sintering, and constraints in achieving very high geometric fidelity. Collectively, these 3D printing approaches enable structural, mechanical, and architectural biomimicry, forming the backbone of contemporary scaffold engineering [84].

#### 3.2.1. Bioprinting of Cell-Based Constructs

Bioprinting enables direct integration of living cells, bioactive hydrogels, and growth factors into precisely defined three-dimensional architectures, achieving biological functionality beyond conventional cell-free scaffolds [22]. This approach allows spatially controlled deposition of mesenchymal stem cells, endothelial cells, and osteoprogenitors, while supporting the formation of perusable vascular-like networks [85]. By incorporating extracellular matrix proteins and tunable hydrogel composites such as Gel MA, alginate, and collagen, bioprinted smart scaffolds deliver hierarchical structural, biochemical, and cellular cues that closely mimic native bone across multiple length scales [86]. Bioinks are frequently formulated with specific cell types such as stem cells, chondrocytes, and fibroblasts to support tissue regeneration among commonly used materials. Gel MA is widely applied in bioprinting due to its biocompatibility and suitability for regenerative medicine [87]. Bioprinting demonstrates high potential for pre-vascularized constructs, osteochondral interfaces, and large-defect reconstructions, but challenges remain in long-term stability and mechanical load bearing [22]. Hydrogel provides a hydrated, biocompatible matrix that supports cell survival and mimics native extracellular environments [88]. Growth factors are incorporated to guide cell differentiation, proliferation, and tissue maturation during and after printing. During extrusion-based bioprinting, a syringe nozzle- mechanism is used to deliver bioinks with controlled flow, supporting accurate layer-by-layer deposition [9,14]. Silvia Baiguera’s study highlights three main bioprinting technologies-inkjet, micro extrusion, and laser-assisted printing. Each technique differs in resolution, cell viability, and compatible biomaterials. Inkjet bioprinting dispenses precise liquid droplets using thermal or piezoelectric actuation and typically maintains high cell viability (>85%). Micro extrusion systems deposit continuous material filaments using pneumatic or mechanical force and support even higher cell viability (~95%), although outcomes depend on gelation kinetics and pressure settings. Laser-assisted bioprinting offers high-resolution patterning without nozzle-induced stress, enabling precise spatial organization of cells and biomaterials in 3D constructs. There are other uses for tissue, organoid, cartilage, and bone constructs. Inkjet bioprinting and laser-assisted bioprinting are two major techniques that differ in how they deposit bioink with precision [89]. These approaches enable the fabrication of early bone microstructure, offering potential for regenerative and orthopedic applications [14].

#### 3.2.2. AI-Guided Scaffold Design and Predictive Optimization

Recent developments in artificial intelligence allow data-driven optimization of scaffold architecture and performance. AI-driven innovation enables generative design of pore geometry and mechanical gradients, prediction of cell migration, nutrient flow, and mechanical failure points, and automated tuning of fabrication parameters and personalized scaffold synthesis based on imaging (CT/MRI). These tools are accelerating the development of patient-specific, high-precision scaffolds and reducing trial and error in material selection, structural design, and mechanical modeling [90].

## 4. Four-Dimensional Printing

Adaptive and responsive 4D printing architectures extend 3D manufacturing by using shape-memory or stimuli-responsive materials that adapt to environmental cues such as temperature, pH, hydration, or mechanical load [30]. Potential advantages include shape adaptation to irregular defect sites, dynamic mechanical reinforcement under load, time-dependent stiffness or porosity adjustments, and controlled release of ions or biomolecules in response to stimuli. These properties mimic bone’s natural capacity to remodel dynamically in response to physiological forces. Although still at an early translational stage, 4D materials represent a key direction for next-generation intelligent scaffolds [91]. Upcoming development should focus on improving mechanical reliability and long-term stability of 4D materials under physiological conditions. Additionally, integrating 4D printing with bioprinting and bioactive material systems will be critical for translating time-responsive scaffolds toward clinically relevant bone regeneration applications.

### Electrospinning and Hybrid Fiber-Based Systems

Electrospinning produces nanofibrous matrices closely resembling the extracellular matrix. These fibers support cell adhesion, migration, and early osteogenic differentiation [92]. Features of electro-spun biomimetic scaffolds include the following: nano-to-microscale fiber control, tunable alignment to guide cell orientation, high surface-area-to-volume ratio, and compatibility with polymer–ceramic composites. Hybrid systems integrating electro-spun layers with 3D-printed frameworks combine macroscale mechanical stability with micro- and nanoscale biomimicry, enhancing osteoconductive surface cues. Such hybrid constructs bridge the gap between structure and function across scales [70].

## 5. Functional Augmentation

Functional augmentation describes strategies used to enhance or introduce new biological, mechanical, or biochemical functions in cells, tissues, or biomaterials to improve their performance [5]. Mechanical augmentation focuses on increasing stiffness, strength, and elasticity so that bone, cartilage, or scaffold constructs can better withstand load [5]. Biological augmentation promotes cell attachment, survival, proliferation, and stem cell differentiation while supporting balanced immune activity. Biochemical augmentation integrates signaling molecules such as BMP-2, TGF-β, cytokines, peptides, or ECM-like components to guide cell behavior [22]. Structural augmentation fine-tunes scaffold pore size, architecture, orientation, and vascular pathways to improve nutrient exchange and tissue integration. Signaling augmentation strengthens immune–vascular–mechanical communication to stimulate osteogenic or chondrogenic pathways [9]. Smart augmentation incorporates stimuli-responsive features that react to pH, temperature, light, or magnetic cues, enabling adaptive or controlled therapeutic responses [45] (Table 2).

### 5.1. Biological Integration into Biomimetic Scaffolds

**Figure 5:**
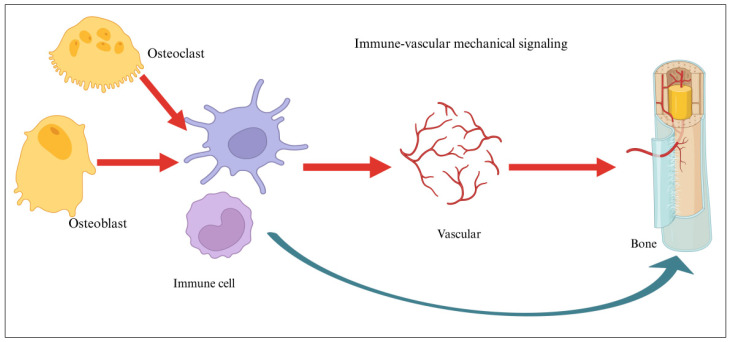
Schematic illustrating osteoimmune vascular coupling in biomimetic bone repair. Osteoblasts, osteoclasts, and monocytes interact with immune cells, which regulate vascular signaling and mechanical stimuli to guide bone remodeling. Biomimetic 3D scaffold integrates multiscale structural features, biochemical ligands, and mechanical cues to modulate immune responses, promote angiogenesis, and enhance osteogenesis, resulting in organized and functional bone regeneration. The feedback loop indicates that bone tissue can influence immune cell activity, highlighting the dynamic interplay between immune signaling, vascularization, and bone regeneration.

Biological integration transforms scaffolds from passive structural templates into active regenerative microenvironments [93]. Incorporating cells, bioactive factors, extracellular matrix (ECM) components, or cell-free vesicles enable scaffolds to emulate the biological intelligence of native bone [94]. The following subsections outline major strategies that enhance scaffold–tissue interactions and guide functional regeneration. Biomimetic bone regeneration employs 3D scaffolds engineered to recapitulate the native bone microenvironment across multiple length scales. At the cellular level, scaffold chemistry and ligand presentation (e.g., RGD peptides and calcium phosphate motifs) regulate osteoblast, osteoclast, and immune cell crosstalk, thereby guiding osteo-immunomodulation. At the microscale, controlled porosity and matrix stiffness support immune–vascular coupling, facilitating angiogenesis and efficient nutrient transport. At the macroscale, scaffold architecture transmits physiological mechanical cues that activate mechano-transduction pathways, synchronizing vascular invasion with new bone formation. Collectively, multiscale structural, biochemical, and mechanical cues coordinate immune signaling, vascularization, and osteogenesis to restore functional bone tissue. The immune vascular interactions act as a conduit through which mechanical forces regulate bone formation [95] (Figure 5).

### 5.2. Cell-Based Strategies for Osteogenic and Vascular Integration

Cell-based strategies enhance bone regeneration by integrating stem and progenitor cells within biomimetic scaffolds to promote osteogenic differentiation and vascular integration. These cells provide both structural contribution and paracrine signaling, supporting coordinated bone formation and neovascularization within engineered constructs [76] (Table 3).

#### 5.2.1. Mesenchymal Stem Cells (MSCs)

Mesenchymal stem cells (MSCs) are a central component of biomimetic bone regeneration strategies because of their dual capacity for osteogenic differentiation and paracrine regulation of inflammation and angiogenesis. When incorporated into smart scaffolds with multiscale hierarchical cues, MSCs display enhanced adhesion, survival, and mineralized matrix depo [96]. Mesenchymal stem cells (MSCs) are a central component of biomimetic bone regeneration strategies because of their dual capacity for osteogenic differentiation and paracrine regulation of inflammation and angiogenesis. When incorporated into smart scaffolds with multiscale hierarchical cues, MSCs display enhanced adhesion, survival, and mineralized matrix deposition. Experimental studies using critical-size bone defect models demonstrate that bone marrow-derived MSCs seeded onto 3D-printed collagen–nano-hydroxyapatite composite scaffolds achieve improved mineral apposition, stronger structural coupling, and accelerated cortical bridging [97]. These findings highlight the significance of combining MSC biology with architecturally and chemically instructive scaffolds; however, gaps remain in achieving consistent vascular integration and long-term functional remodeling, underscoring the need for more predictive and translational scaffold cell systems.

#### 5.2.2. Adipose-Derived Stem Cells (ADSCs)

Adipose-derived stem cells (ADSCs) are increasingly recognized as an effective cell source in biomimetic bone regeneration due to their high availability and strong pro-angiogenic activity [98]. When integrated into smart scaffolds based on Gel MA, bioactive glass hybrids, or composite hydrogels with multiscale cues, ADSCs promote vascular sprouting and support early defect bridging in preclinical bone defect models [99]. Adipose-derived stem cells (ADSCs) are increasingly recognized as an effective cell source in biomimetic bone regeneration due to their high availability and strong pro-angiogenic activity.ey findings demonstrate enhanced neovascularization and improved early tissue integration, highlighting their significance for accelerating regeneration. However, compared with bone marrow-derived MSCs used in 3D-printed collagen–nano-hydroxyapatite scaffolds that achieve robust mineral apposition and cortical bridging in critical-size defects, gaps remain in optimizing long-term osteogenic stability and mechanical maturation of ADSC-based constructs, indicating the need for refined scaffold designs and combinatorial cues.

#### 5.2.3. Induced Pluripotent Stem Cell-Derived Osteoprogenitors (iPSC-OPs)

Induced pluripotent stem cell-derived osteoprogenitors (iPSC-OPs) represent a highly promising, patient-specific, and scalable cell source for biomimetic bone regeneration. In experimental models, iPSC-OPs are integrated into smart scaffolds with multiscale cues such as nanofibrous PCL/collagen architectures combined with Gel MA matrices incorporating hydroxyapatite nanoparticles, which exhibit synergistic responses to mechanical and biochemical signals. Key findings demonstrate enhanced mineralization, improved immune compatibility, and coordinated interaction with developing vascular networks, underscoring the significance of iPSC-OP-based smart scaffold systems for personalized bone modeling and regenerative repair [100].

#### 5.2.4. Periosteum-Derived Cells (PDCs)

Periosteum-derived cells (PDCs) play a critical role in biomimetic bone regeneration strategies due to their inherent osteogenic and immunomodulatory functions. In experimental studies, PDCs integrated into smart scaffolds composed of silk fibroin, polycaprolactone (PCL), and nano-hydroxyapatite, with growth factor-functionalized surfaces, demonstrate rapid periosteal integration and continuous osteoid formation. These multiscale scaffold cues effectively replicate the native periosteal envelope, resulting in improved load transfer and accelerated cortical bone regeneration, highlighting the significance of PDC-based smart scaffold systems for functional bone repair [101].

#### 5.2.5. Co-Culture Systems

Co-culture systems represent an advanced biomimetic strategy for bone regeneration by integrating osteogenic and vascular cell populations within smart scaffolds to replicate native osteo–vascular coupling. In bioprinter or composite constructs such as layer-by-layer-assembled Gel MA/alginate matrices combined with type I collagen and β-tricalcium phosphate spatially organized endothelial cell MSC co-cultures form pre-vascularized micro-niches [22]. Experimental findings demonstrate accelerated inosculation with host vessels, reduced necrotic regions, enhanced neovascularization, and improved long-term graft viability and integration, underscoring the importance of multiscale scaffold cues for functional and stable bone regeneration [102].

#### 5.2.6. Biomimetic Hydrogel Platforms Supporting Osteoblastic Function

Osteoblast-laden PEG–HA hydrogels represent a smart, biomimetic hydrogel platform for bone regeneration by providing a cell-instructive microenvironment with multiscale cues. Formed through photo-crosslinked polyethylene glycol networks functionalized with hyaluronic acid, these hydrogels preserve osteoblastic phenotypes while offering mechanical stability and HA-mediated cell matrix signaling [77]. This synergistic combination promotes organized lamellar bone deposition and has demonstrated effective performance in craniofacial defect models, highlighting the significance of PEG–HA hydrogels as functional smart scaffolds for bone regeneration [14].

#### 5.2.7. Expose-Loaded MSC Scaffolds for Immunomodulatory Bone Regeneration

Exosome-loaded MSC scaffolds constitute an advanced biomimetic strategy that integrates immunomodulatory signaling into smart, multiscale bone regeneration platforms. Porous PLGA–caprolactone matrices reinforced with bio glass–collagen composites provide mechanical support while enabling the controlled delivery of MSC-derived exosomes that regulate the early inflammatory phase. This immune-responsive scaffold microenvironment promotes balanced inflammation, accelerates osteogenic maturation, and supports rapid early remodeling, underscoring the significance of exosome-based smart scaffolds for functional and stable bone regeneration [103] (Table 4).

### 5.3. Bioactive Factor Integration

Integrating living cells and bioactive signals transforms scaffolds from inert supports into regenerative microenvironments. Stem cell incorporation provides biological intelligence for tissue repair [104]. Mesenchymal stem cells (MSCs) and adipose-derived stem cells (ADSCs) enhance osteogenesis and angiogenesis through paracrine secretion [105]. Induced pluripotent stem cells (iPSCs) offer limitless expansion and patient-specificity, while periostea-derived progenitors exhibit superior osteogenic potential and immune compatibility [106]. Controlled release systems sustain local delivery of growth factors and peptides, thereby maintaining spatiotemporal signaling. Gradual release of BMP-2, VEGF, or SDF-1 directs sequential osteogenic and vascular events, while ECM-derived peptides improve cell adhesion and differentiation [107]. Exosome-based strategies have emerged as a cell-free alternative, mediating regenerative communication through miRNAs and proteins that activate osteo-angiogenic pathways. Decellularized matrices provide a natural template rich in native cues, supporting cell attachment and lineage-specific remodeling [33]. Recent studies highlight synergistic effects when combining stem cells with exosomes or decellularized scaffolds, resulting in enhanced vascularized bone formation [108].

### 5.4. Bioactive Factors and Controlled Delivery Systems

Biomimetics integrate bioactive factors to guide cell behavior and enhance regenerative signaling. Controlled release systems ensure that these cues are delivered in a timed and dose-appropriate manner that mirrors natural bone healing processes [109]. Such engineered delivery strategies improve tissue maturation, vascularization, and overall repair outcomes. In a clinical study, Tiffany N. Vo et al. investigated ECM-Hap delivery system in which Rat calvaria osteoblast and dermal fibroblast and Rat calvaria defect; compared to Hap control, it exhibited greater integrated bone formation, no complete bridging, or difference between types of cell-generated ECM.

### 5.5. Growth Factor Delivery

BMP-2 drives strong osteogenic differentiation while VEGF promotes vascular ingrowth, essential for graft survival. SDF-1 enhances the endogenous for stem cell recruitment and supports early regenerative events [22]. Sequential delivery systems replicate natural healing timelines and improved coordinated tissue responses. Advanced carriers such as microspheres, nanogels, and mineral release systems using microspheres, nanocarriers, hydrogels, or mineral coatings minimize burst release and maintain sustained local signaling [110]. In a recent study by Tiffany N. Vo et al., in vivo applications of collagen materials by BMP-2 growth factors have been demonstrated, whereas apatite-coated scaffold in mouse calvaria CSD apatite coating prolonged BMP activity, synergistic enhancement of bone formation, and mineralization.

### 5.6. Ion-Based Bioactivity

Bioactive ions including Mg^2+^, Sr^2+^, Zn^2+^, Si^4+^, and Ca^2+^ act as signaling mediators that upregulate osteogenic genes, supporting angiogenesis and regulating immune responses. They are control-release creations into a pro regenerative microenvironment that accelerates bone formation and tissue integration [5].

### 5.7. ECM-Derived Peptides

ECM-derived peptides provide cells with precise adhesion and signaling cues. Motifs such as RGD and GFOGER enhance integrin engagement and support stable osteoblast attachment. Collagen-mimetic sequences further reinforce matrix organization and lineage-specific activity [111]. These peptides strengthen osteoconductive and osteo inductive responses within the scaffold. They also improve early angiogenic signaling, promoting more efficient tissue integration.

### 5.8. Exosome-Based and Cell-Free Regenerative Strategies

Exosomes and extracellular vehicles (EVs) have emerged as powerful mediators of intercellular communication, delivering microRNAs, proteins, and lipids that regulate osteogenesis, angiogenesis, and immune responses [112]. Exosome-loaded scaffolds offer several advantages. They avoid the risks associated with live-cell transplantation, provide sustained release of therapeutic cargo, and exhibit low immunogenicity [98]. They also promote synchronized osteogenic and angiogenic activation [14]. MSC- or immune cell-derived exosomes integrated into porous scaffolds have demonstrated improved defect healing and reduced inflammation in rodent models [113].

### 5.9. Decellularized ECM-Based Platforms

Decellularized extracellular matrix (DECM) retains native structural proteins, growth factors, and biochemical motifs that are difficult to reproduce synthetically [77]. Benefits of DECM integration are as follows: it provides a natural microenvironment for osteogenic lineage guidance, enhances early cell adhesion and migration [58], offers species-specific biochemical signals, and supports mineral nucleation and matrix deposition [3,45]. ECM-based scaffolds have demonstrated superior remodeling and mechanical durability in large-animal preclinical models. Hybrid composites combining DECM with synthetic polymers further improve consistency, mechanical strength, and translational viability [114].

### 5.10. Synergistic Integration of Biological and Structural Cues

The most effective biomimetic scaffolds combine multiscale architecture with biological components. For example, hierarchical 3D frameworks and MSCs enhance defect bridging and bioactive ceramic composites; growth factor gradients promote osteo–vascular coupling; and DECM layers and iPSC-OPs support lamellar bone formation [82]. Hybrid electro-spun-printed constructs and exosomes improve immune regulation. These synergistic platforms mimic both the physical and biological environment of native bone, enabling more predictable and durable regenerative outcomes [115].

### 5.11. In Vitro Optimization of Biomimetic Scaffolds

In vitro assays offer controlled platforms to evaluate the fundamental behavior of biomimetic scaffolds. They allow precise monitoring of cell adhesion, proliferation, and early differentiation responses. Mechanical and degradation testing further clarifies scaffold stability and functional suitability [116]. These evaluations help refine material composition and architecture before animal implantation. Overall, in vitro optimization provides essential evidence supporting progression toward preclinical validation [117].

### 5.12. Osteogenic and Mechanobiological Evaluation

Engineered scaffolds must support osteoblast and MSC adhesion, proliferation, and matrix deposition. They should promote the upregulation of key osteogenic markers, including RUNX2, ALP, OCN, and COL1A1. Successful scaffolds must also enable mineralization under static or dynamic culture conditions [118]. Scaffolds should generate physiologically relevant mechano-transduction responses. Mechanical stimuli, such as cyclical loading, fluid shear stress, or piezoelectric activation, are essential for evaluating mechanoresponsive materials and predicting in vivo remodeling [119].

### 5.13. Immune and Inflammatory Profiling

Immune compatibility is assessed by macrophage phenotype modulation (M1 → M2 transition), cytokine secretion profiles (TNF-α, IL-6, IL-10, TGF-β), and foreign-body response quantification. Materials promoting early inflammatory resolution and constructive remodeling are prioritized for translation [120].

### 5.14. Angiogenic and Endothelial Performance

Angiogenic and endothelial performance is assessed through multiple in vitro models that simulate early vascular development. Key methods include endothelial tube formation assays, perusable microchannel platforms, and osteogenic co-culture systems. These models provide insight into vessel-maturation cellular interaction and functional vascular coupling, perusable microchannel modeling, and co-culture systems mimicking osteo- and vascular coupling. Collectively, they help predict early perfusion and long-term graft integration [121].

## 6. Incomplete Reproduction of Native Bone Complexity

Bone is a dynamic, multiscale tissue with synchronized structural, mechanical, immune, and vascular functions. Even the most advanced biomimetic scaffolds struggle to simultaneously replicate cortical–trabecular mechanical gradients and a multiscale nano–micro–macro hierarchy [78], as well as spatially and temporally regulated biochemical signaling, region-specific immunomodulatory responses, and interconnected vascular networks. Most scaffold systems capture only selected components of this complexity. The inability to fully emulate native osteo–vascular and osteoimmune coupling remains a principal barrier to robust clinical performance [75].

### 6.1. Biological Variability and Uncertain Host Response

Host-specific biological variability complicates scaffold performance. Key sources of variability are patient age, metabolic status, and osteoporotic changes; variation in immune sensitivity and inflammatory tone [122]; differences in bone turnover, vascular density, and marrow physiology; and donor-dependent heterogeneity in stem cells or exosomes [77]. These factors can produce unpredictable outcomes, particularly for cell-based or immunomodulatory scaffolds. Without precise patient stratification or predictive biomarkers, therapeutic efficacy may vary widely.

### 6.2. Mechanical Stability and Long-Term Reliability

While many scaffolds demonstrate promising osteogenesis in small animals, load-bearing mechanical performance remains a challenge in humans. Major mechanical limitations include insufficient strength for large or high-load defects, fatigue failure under cyclic loading [123] mismatch in stiffness causing stress shielding, degradation rates that are either too rapid or too slow, and limited long-term data on adaptive or 4D materials. Achieving clinically reliable mechanical performance requires robust validation in large animals and under physiological loading conditions.

### 6.3. Vascularization Bottlenecks

Early vascularization is essential for implant viability, yet achieving rapid, stable, and perusable vasculature remains difficult. Persistent limitations include slow inosculation with host vessels, immature or unstable microvasculature in engineered constructs, necrotic cores in large, dense scaffolds, and oxygen diffusion constraints in thick 3D or bio-printed tissues [33]. These constraints directly affect scaffold survival and long-term remodeling [124].

### 6.4. Scalability and Manufacturing Challenges

Biomimetic scaffolds involve complex fabrication processes that may not scale efficiently. High-resolution 3D/4D printing is time- and resource-intensive, and multi-material or cell-laden systems require a stringent sterile environment. Batch-to-batch reproducibility is difficult for hybrid or biologically functionalized scaffolds. Biomolecule incorporation increases regulatory classification complexity [33]. DECM-based materials may face raw material shortages and variability. Transitioning from laboratory prototypes to industrial-scale GMP production remains a major hurdle [43].

### 6.5. Regulatory and Safety Constraints

The regulatory pathway for biomimetic scaffolds is often unclear, especially for hybrid or smart constructs that combine materials, cells, exosomes, or adaptive responses [119]. Key regulatory challenges are classification as Class III medical devices or advanced therapy medicinal products (ATMPs), stringent requirements for sterility, long-term safety, and functional stability, need for standardized testing for immune modulation and smart material behavior, limited precedent for approval of 4D or bioresponsive scaffolds, uncertainty in long-term degradation, and biosafety profiles. These constraints slow clinical adoption and increase development costs [46].

### 6.6. Cost and Accessibility Barriers

The economic feasibility of biomimetic scaffolds is still uncertain. Advanced manufacturing, cell-based components, and GMP processing significantly increase costs. Challenges include high production and regulatory compliance costs, limited reimbursement frameworks for regenerative implants, a lack of standardized cost-effectiveness data, and scalability requirements for widespread clinical use [78]. Without cost optimization and clear reimbursement pathways, clinical adoption will remain limited [122].

#### 6.6.1. In Situ Bioprinting and On-Site Regeneration

In situ bioprinting enables the direct deposition of bioinks, cells, or composites into the defect site, conforming precisely to the native geometry without the need for prefabricated implants [2]. Advantages include custom-fit repair of irregular or large defects, real-time integration with host tissue microenvironment, minimization of surgical manipulation and graft mismatch, and potential for on-demand delivery of cells, growth factors, and exosomes. Portable bioprinting systems and robotic-assisted deposition are actively being developed, yet challenges remain in stabilizing printed constructions in vivo and ensuring rapid vascularization [10].

#### 6.6.2. Smart and Responsive Scaffold Systems

Future biomimetic scaffolds will increasingly incorporate real-time sensing, adaptive behavior, and feedback-controlled responses [99]. Key directions are as follows: stimuli-responsive materials (temperature, pH, strain, and electrical signals), adaptive mechanical systems that stiffen or soften underload, electrically conductive or piezoelectric scaffolds for bioelectric stimulation [76], metabolite-responsive release of therapeutic molecules, embedded micro- or nanosensors for monitoring perfusion, pH, inflammation, or mechanical stress. These platforms aim to mimic bone’s dynamic responsiveness, accelerating remodeling and improving integration [124].

#### 6.6.3. Bone Organoids, Organ-on-Chip Platforms, and Advanced Disease Modeling

Organoid and micro physiological systems offer powerful tools for accelerating preclinical research [52]. Potential applications are as follows: modeling osteogenesis, angiogenesis, and osteoimmune interactions, high throughput testing of scaffold materials and drug combinations [9], predicting patient-specific responses using iPSC-derived bone organoids, studying pathologies such as osteoporosis, osteomyelitis, or cancer-associated bone loss, and reducing reliance on small animal models for early testing. Bone-on-chip platforms that integrate flow dynamics, immune components, and mechanical loading may become essential tools for scaffold optimization [53].

#### 6.6.4. Personalized Regenerative Implants

The convergence of imaging, 3D printing, and AI enables patient-specific implants tailored to individual defect geometry, bone quality, and biological profile [51]. Frontiers include preoperative CT-based modeling of defect mechanics, personalized pore architecture for variable bone density, AI-based prediction of optimal scaffold stiffness, porosity, and degradation, and tailored biochemical and immunomodulatory profiles based on patient-specific biomarkers. These advances promise to address major sources of variability in regenerative outcomes [49].

#### 6.6.5. AI-Driven Closed-Loop Design and Automated Optimization

Artificial intelligence will increasingly automate scaffold design, predictive modeling, and experimental optimization. Key opportunities include generative scaffold design based on functional requirements, predictive modeling of osteogenesis, vascularization, and mechanical performance, and automated parameter tuning for 3D/4D printing [34]. Digital twins will enable real-time monitoring and adaptation of scaffold behavior [54]. Machine learning integration with organoid or organ-on-chip outputs will further enhance biological relevance [56]. Closed-loop systems that refine scaffold design through iterative biological feedback represent a transformative step toward personalized regenerative medicine [51].

#### 6.6.6. Integrating Multi-Omics and Precision Biomaterials

Genomic, proteomic, metabolomic, and glycemic data offer insights into patient-specific regenerative biology [46]. Prospects are as follows: biomaterial tuning based on osteoimmune signatures, exosome or EV loading tailored to patient transcriptomic profiles, ion-release systems designed for metabolic deficiencies, and personalized risk stratification for scaffold selection. This integration will enable high-precision, biologically matched biomaterials [48,57].

#### 6.6.7. Scalable GMP Production and Regulatory Harmonization

Clinical translation requires not only scientific innovation but also manufacturing feasibility and regulatory alignment [43]. Required progress is as follows: cost-efficient high-resolution printing technologies, standardized sterilization and storage for biologically functional scaffolds, modular GMP facilities for hybrid and cell-integrated systems, regulatory guidance for smart, adaptive, or AI-driven implants, and long-term surveillance systems for biosafety and mechanical reliability. Collaboration among industry, academia, and regulatory agencies will be essential [63] (Table 5).

#### 6.6.8. Regulatory Considerations

Biomimetic scaffolds are advancing from bench to bedside in complex orthopedic settings [42,111]. Preclinical and early clinical reports demonstrate enhanced union rates and reduced graft failure [112]. Spinal fusion applications utilize bioactive and mechanically graded scaffolds that promote osteointegration while maintaining stability. Incorporation of osteoinductive factors has shown promising fusion outcomes in animal and pilot human trials [109].

## 7. Limitations and Future Directions

**Figure 6:**
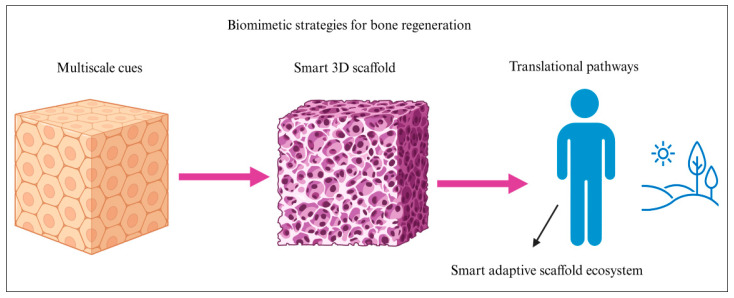
Schematic illustration shows the integration of multi-scale biomimetic cues into a smart 3D scaffold for bone regeneration. Nano- and microscale ECM mimetic features provide biochemical and mechanical signals that regulate cell adhesion, proliferation, and osteogenic differentiation, while macroscale scaffold architecture ensures structural support and defect conformity. The smart scaffold dynamically adapts to the biological environment through controlled degradation and signaling, ultimately enabling functional bone regeneration and translational applications.

Despite substantial progress in materials science, biofabrication, and regenerative biology, biomimetic scaffolds continue to face significant scientific, manufacturing, and regulatory constraints [113]. These limitations must be understood critically to enable meaningful clinical translation [114]. Continued innovation in biomimetic scaffold engineering is rapidly expanding the boundaries of bone regeneration [74]. Future research must focus on integrating multiscale biological insights with intelligent material systems, precision design tools, and clinically feasible manufacturing pathways [115]. The following directions are poised to define the next generation of regenerative technologies [76]. Even with significant progress, biomimetic design and smart fabrication cannot yet reproduce the full biological and mechanical dynamics of natural bone tissue [116]. Challenges such as high production cost, limited scalability, and unpredictable cell-tissue responses still restrict their smooth transition into clinical use [17,33]. Long bone fractures may require stable microporous scaffolds, while flat bones could benefit from microporous scaffolds with controlled pore sizes [79]. Customized scaffold designs tailored to specific pore structures and material properties may be necessary for optimal healing [117]. Researchers should carefully consider these factors and conduct comprehensive studies to assess scaffold effectiveness across different bone types and anatomical locations [118]. In situ bioprinting is emerging as a surgical technology, allowing the direct deposition of living bioinks into defect sites for seamless tissue integration and reduced operative time [119]. Smart scaffolds equipped with biosensors and feedback circuits can monitor local pH, strain, or inflammatory cues, releasing therapeutic molecules in real time to guide healing. The figure illustrates how biomimetic porous scaffolds, enriched with targeted bioactive cues, can restore bone function and ultimately support a return to healthy mobility and quality of life. Multiscale structural cues embedded within smart biomimetic scaffolds, ranging from nano- to macroarchitecture, recapitulate native bone hierarchy and regulate cell–material interactions, where ECM-like ligands and scaffold stiffness modulate integrin-mediated mechano-transduction, activating osteogenic signaling pathways such as Runx2,BMP/Smad, and Wnt/β-catenin, while adaptive scaffold functions including controlled degradation, growth factor delivery, and load responsiveness support coordinated osteogenesis, angiogenesis, and progressive remodeling into mature bone tissue [73] (Figure 6).

## 8. Conclusions

Biomimetic and smart fabrication strategies are rapidly transforming bone scaffold design from passive structures into intelligent, adaptive platforms. By integrating multiscale architectural cues with dynamic immune, vascular, and mechanical feedback, emerging systems more closely reproduce the functional complexity of native bone. Continued advances in AI-guided design, organoid-based evaluation, and programmable biomaterials will accelerate the development of responsive, patient scaffolds. Together, these innovations mark a decisive step toward regenerative intelligence in skeletal medicine. Biomimetic scaffold engineering has progressed from simple templates to multifunctional, biologically integrated systems. Smart fabrication technologies now enable precise control over architecture and microenvironmental cues. Advances in 3D/4D printing, electrospinning, and bioprinting support highly tunable designs. Parallel developments in immunomodulation, ECM-derived motifs, and exosome therapeutics enhance biological signaling. Despite these gains, replicating bone’s full hierarchical organization remains challenging. Dynamic immune regulation and stable vascular integration remain difficult to achieve. Mechanical reliability and manufacturing scalability further limit clinical translation. Multi-disciplinary collaboration is essential to overcome these barriers. AI-guided design and organoid-based testing are shaping the next generation of scaffolds. In situ fabrication and precision biomaterials will enable patient-specific solutions. These innovations are transforming static construction into adaptive, responsive platforms. Such convergence marks a decisive step toward regenerative intelligence in skeletal medicine.

## Figures and Tables

**Table 1:** Fabrication technologies for biomimetic bone scaffolds: biological strength, limitations, and clinical readiness.

Technology	Resolution	Suitable Materials	Biological Strength	Clinical Readiness	Limitations	References
Fused Deposition Modeling (FDM)	100–300 µm	Thermoplastics (PCL, PLA, PLGA)	Good mechanical integrity, limited bioactivity	High FDA-approved polymers, used in craniofacial repair	Limited resolution for micro-vascular and cell-laden constructs	[14,41]
Stereolithography (SLA)	<50 µm	Photocurable resins, ceramic polymer hybrids	Excellent structural accuracy, tunable stiffness	Moderately improving biocompatibility of resins	Cytotoxicity risk due to unpolymerized resin and UV exposure	[63]
Selective Laser Sintering (SLS)	50–200 µm	Metals, ceramics, polymer composites	High strength, suitable for load-bearing implants	High clinical prototypes for long bone defects	High processing temperature prevents direct cell incorporation	[14]
Electrospinning	<500 nm fiber diameter	Collagen, Gel MA, PCL, silk fibroin	Mimics ECM nanofibers and supports cell adhesion	Moderately used in guided bone regeneration	Poor mechanical stability and limited scaffold thickness	[64]
Bioprinting	50–200 µm	Hydrogels, bioinks, cell-laden matrices	High biological functionality and vascularization potential	Emerging validated in preclinical craniofacial models	Lower mechanical strength and slow printing compared to polymer extrusion	[9]
4D Printing	Variable, shape-adaptive	Shape-memory polymers and composites	Dynamic and stimuli-responsive remodeling	Early translational stage	Limited material availability and low long-term stability data	[65]

**Table 2:** Types of biomimicry in bone scaffold design.

Type of Biomimicry	Design Strategy	Recent Examples	Observed Outcomes	References
Structural Biomimicry	Gradient porosity, oriented microchannels, hierarchical layering	3D-printed hydroxyapatite/gelatin scaffolds mimicking cortical trabecular transitions	Enhanced mechanical strength, guided cell infiltration, improved osteoconduction	[17]
Biochemical Biomimicry	Incorporation of ECM peptides, ions, or controlled release of BMP-2, VEGF	Ion-doped bioactive glass with sequential BMP-2/VEGF delivery	Coupled osteogenesis and faster mineralization	[45]
Mechanical Biomimicry	Mechanoresponsive or piezoelectric polymers converting stress into bioelectric signals	Polyvinylidene fluoride (PVDF)-based piezoelectric scaffold under cyclic loading	Stimulated osteoblast differentiation via Wnt/β-catenin activation	[45]
Immune Biomimicry	Modulation of macrophage polarization (M2) via surface chemistry or ion release	Zn–Mg-modified bioceramics promote M2 phenotype	Reduced inflammation, accelerated vascularized bone formation	[75]
Vascular Biomimicry	Pre-vascularized channels, angiogenic factor gradients	Bio-printed Gel MA scaffolds with endothelial co-culture	Improved perfusion, rapid integration with host	[74,76]
Integrated Biomimicry	Sensing feedback systems, AI-guided adaptive designs	4D-printed shape-memory composite with biosensing microchips	Real-time response to mechanical/chemical cues, adaptive bone remodeling	[76]

**Table 3:** This table presents a comparative overview of scaffold materials and key functional characteristics.

Cell Source	Scaffold Types	Material Composition	Integration Strategy	Key Outcome	Recent Application	References
Bone Marrow-Derived MSCs (BM-SCs)	3D-printed composite	Type I collagen matrix reinforced with nano-hydroxyapatite	Direct cell seeding with osteogenic priming	Enhanced mineral apposition and structural coupling	Accelerates cortical bridging in critical-size defects	[74]
Adipose-Derived Stem Cells (ADSCs)	PCL– Gel MA bioactive glass hybrid	Polycaprolactone framework with GelMA hydrogel coating	3D bioprinting within gradient pores	Improved angiogenic index and increased bone density	Demonstrated synergistic vascular–osteogenic interface	[79]
Induced Pluripotent Stem Cell-Derived Osteoprogenitors (iPSC-OPs)	Nanofibrous PCL/collagen scaffold	Gelatin methacrylate with dispersed hydroxyapatite nanoparticles	Encapsulation under dual mechanical–biochemical cues	Superior osteoconductivity with immune compatibility	Enables patient-specific bone modeling and repair	[79]
Periosteum-Derived Cells (PDCs)	Silk fibroin/n HA composite	Silk proteins blended with polycaprolactone fiber mesh	Growth factor surface immobilization	Rapid periosteal integration and osteoid continuity	Biomimicry of periosteal envelope supports improved load transfer	[79]
Endothelial–MSC Co-culture	Bioprinter Gel MA/alginate construct	Type I collagen matrix combined with β-tricalcium phosphate granules	Layer-by-layer engineered assembly	Formation of vascularized micro-niches and faster inosculation	Proven in vivo to reduce necrotic zones and improve graft viability	[14]
Osteoblast-Laden Hydrogel	PEG–HA hydrogel	Polyethylene glycol network functionalized with hyaluronic acid	Photo crosslinked encapsulation	Maintains osteoblastic phenotype and promotes lamellar bone deposition	Applied in craniofacial defect scaffolds	
Exosome-Loaded MSC Scaffold	Porous PLGA/Cap matrix	Bioglass particulates embedded within a collagen matrix	Immunomodulatory preconditioning	Balanced inflammatory cascade with accelerated bone maturation	Demonstrates immune–scaffold synergy supporting early remodeling	[80]

**Table 4:** Biomimetic scaffold strategies for bone regeneration: study focus, experimental models, and key outcomes.

Biomimetic Strategy	Study Focus Mechanistic Target	Experimental Model	Key Findings	Reference
Collagen–Hydroxyapatite (Col-HA) Composite	Mimic native bone mineral–collagen alignment for osteoconductive support	Rat critical-sized calvaria defect	Enhanced new bone formation and vascularization; upregulated Runx2 and OCN expression	[14,87]
BMP-2 Loaded HA-Nanofiber Scaffold	Controlled spatiotemporal release of osteoinductive cues	Rabbit femoral defect model	Accelerated defect bridging; increased trabecular density and biomechanical strength	[3]
Bioactive Glass with Immunomodulatory Surface Patterning	Shift macrophage phenotype from M1 (pro-inflammatory) to M2 (pro-healing)	Mouse subcutaneous implantation model	Reduced inflammatory cytokines; enhanced osteogenesis through OSM and TGF-β1 signaling	[14]
3D Printed Gradient Scaffold (Stiffness + Microarchitecture)	Replicate zonal biomechanics and hierarchical porosity	Sheep tibial load-bearing defect	Improved load transfer, mineralization, and integration at host tissue interface	[88]
ECM-Mimetic Peptide Functionalized Hydrogel	Enhance cell adhesion and early progenitor recruitment via integrin signaling	In vitro MSC culture + rat defect	Increased focal adhesion kinase signaling, early osteogenic differentiation, and matrix deposition	[89]

**Table 5:** Biomimetic scaffold matrices for bone regeneration: composition, function, and research outlook.

Matrix Type	Composition/Example	Structural Traits	Biofunction and Strength	Research Outlook	References
Natural Polymer Matrix	Collagen, Gel MA, Chitosan	Native-like fibrillar network; tunable elasticity	Enhance cell adhesion, early osteoid formation; moderate load bearing	Favored for hybrid scaffold fabrication, it requires mechanical fortification	[13]
Synthetic Polymer Matrix	PLGA, PCL, PEG-based blends	Uniform micro-porosity; programmable degradation	Enables spatiotemporal growth factor release; high reproducibility	Extensively studied, biodegradable kinetics under optimization	[106]
Ceramic-based Matrix	Hydroxyapatite (HA), β-TCP	Rigid crystalline lattice; osteoconductive surface	Supports mineral nucleation; mimics bone inorganic phase	Widely used in defect filling; brittleness limits large defects	[60]
Composite Matrix	n HA Collagen, PCL–β-TCP	Hierarchical microarchitecture; balanced stiffness	Combines toughness with bioactivity; promotes angiogenesis	Trending in next-gen bone grafts; translation under regulatory review	[3]
Decellularized ECM	Bone/Cartilage-derived ECM	Preserved natural ultrastructure; species-specific	Maintains signaling peptides; enhances stem cell fate guidance	Increasing clinical relevance; batch variability persists	[107]
Hydrogel Matrix	Alginate, Fibrin, Gel MA -HA hybrid	Hydrated viscoelastic mesh; injectability	Sustains viable cell encapsulation; suitable for minimally invasive delivery	Used in bioprinting; mechanical fatigue under study	[74]
Bioactive Glass Matrix	Si O_2_–CaO–P_2_O_5_ systems	Ionic dissolution-driven remodeling	Triggers osteogenic gene expression; modulates immune tone	Advanced craniofacial repair; compositional tuning ongoing	[108]

## Data Availability

Data will be made available on request.
